# Longitudinal changes in physical activity, sedentary behavior and body mass index in adolescence: Migrations towards different weight cluster

**DOI:** 10.1371/journal.pone.0179502

**Published:** 2017-06-21

**Authors:** José Devís-Devís, Jorge Lizandra, Alexandra Valencia-Peris, Esther Pérez-Gimeno, Xavier García-Massò, Carmen Peiró-Velert

**Affiliations:** 1 Departament d’Educació Física i Esportiva, Universitat de València, Valencia, Spain; 2 Departament de Didàctica de l’Expressió Musical, Plàstica i Corporal, Universitat de València, Valencia, Spain; Dartmouth College Geisel School of Medicine, UNITED STATES

## Abstract

This study examined longitudinal changes in physical activity, sedentary behavior and body mass index in adolescents, specifically their migrations towards a different weight cluster. A cohort of 755 adolescents participated in a three-year study. A clustering Self-Organized Maps Analysis was performed to visualize changes in subjects’ characteristics between the first and second assessment, and how adolescents were grouped. Also a classification tree was used to identify the behavioral characteristics of the groups that changed their weight cluster. Results indicated that boys were more active and less sedentary than girls. Boys were especially keen to technological-based activities while girls preferred social-based activities. A moderate competing effect between sedentary behaviors and physical activities was observed, especially in girls. Overweight and obesity were negatively associated with physical activity, although a small group of overweight/obese adolescents showed a positive relationship with vigorous physical activity. Cluster migrations indicated that 22.66% of adolescents changed their weight cluster to a lower category and none of them moved in the opposite direction. The behavioral characteristics of these adolescents did not support the hypothesis that the change to a lower weight cluster was a consequence of an increase in time devoted to physical activity or a decrease in time spent on sedentary behavior. Physical activity and sedentary behavior does not exert a substantial effect on overweight and obesity. Therefore, there are other ways of changing to a lower-weight status in adolescents apart from those in which physical activity and sedentary behavior are involved.

## Introduction

Physical activity (PA), sedentary behavior (SB) and weight status are a triumvirate of variables that has attracted much of the recent research on children and adolescents, due to their health consequences. The increases in the prevalence of overweight and obesity in children and adolescents [1,2] is widespread. Similarly, it is known that PA and SB patterns are determinant in the weight status of youth [3–5].

Several longitudinal studies support that PA decreases with age [6,7] whereas SB increases as adolescents get older [8,9]. Since PA has energy expenditure associated [10] and SB reduces energy consumption [11], weight status is often known as the result of an energy imbalance [12] and a prevailing SB time upon time devoted to PA is usually associated with weight gain [7]. According to Must and Tybor’s review [13] of longitudinal studies on weight and adiposity in youth, an increase in time spent on PA and a decrease in SB protected against relative weight and fat gain during these years. However, there are studies that bring some counterintuitive ideas about the triumvirate’s relationships. Collings et al. [14] found that larger increase of body fat appeared in adolescents who reported being more active. Other studies report that, although PA has some influence on body mass index (BMI), results tend to show weak relationships between PA and overweight status and obesity [15,16]. There is even a study that questions whether sports programs protect youth from becoming overweight or obese [17].

Research evidence suggests that overweight and obesity are strongly associated with SB, particularly with screen media usage (SMU) [18,19], and even more than PA [20]. Nonetheless, there are studies that found no ties between SMU and the risk of being overweight or obese in youth [21,22], nor that depended on the type of SB [23]. While TV viewing is a SB often related with being overweight [24], other leisure or academic activities, such as playing videogames, using computers or reading, seem not having clear effect on weight [25].

Regarding gender differences, research indicates that boys are more active than girls, who also tend to be more sedentary [26]. However, while increased hours spent in SMU is associated with being overweight or obese in both boys and girls who do not comply with PA guidelines, it is only significant regarding physically active girls [27]. Gender differences in SB indicate that boys devote more time to computer and videogames usage [28] while girls spend more time on social-leisure sedentary activities, such as meeting with friends or using mobile phones for social-communication [8].

Although linear statistical models have been employed to assess the relation among PA, SB and weight, it would be desirable to use other innovative methods to explain the complexity of the biological and social dimensions of these variables, as different aspects of the adolescents’ lifestyle. Hence, following Laurson et al. [29], we suggest working with the triumvirate of variables through an alternative analysis, such as Self-Organizing Maps (SOM), commonly used to classify and display both, the linear and non-linear relationships among variables.

The purpose of this paper is to examine longitudinal changes in PA, SB and BMI in Spanish adolescents, specifically focusing on adolescents who migrate towards a lower weight cluster. In order to achieve such a purpose, two concatenated phases are developed. Initially, a clustering SOM analysis is performed to understand interrelationships among PA, SB and BMI, and how adolescents are grouped. SOM is a non-linear model of analysis especially adequate to visualize and establish topological links among complex interrelationships, such as studied in this paper. The second phase is developed to quantify adolescents who migrate from one cluster to another in a three-year time period and to identify, through a classification tree, the behavioral characteristics of the groups that change towards a different weight cluster.

## Materials and methods

### Ethics statement

The individual schools, the school districts and the Ethics Committee of the Universitat de València approved materials and procedures used in this study. Parents and participants aged 18 years or over provided written informed consent forms.

### Participants

**Fig 1:**
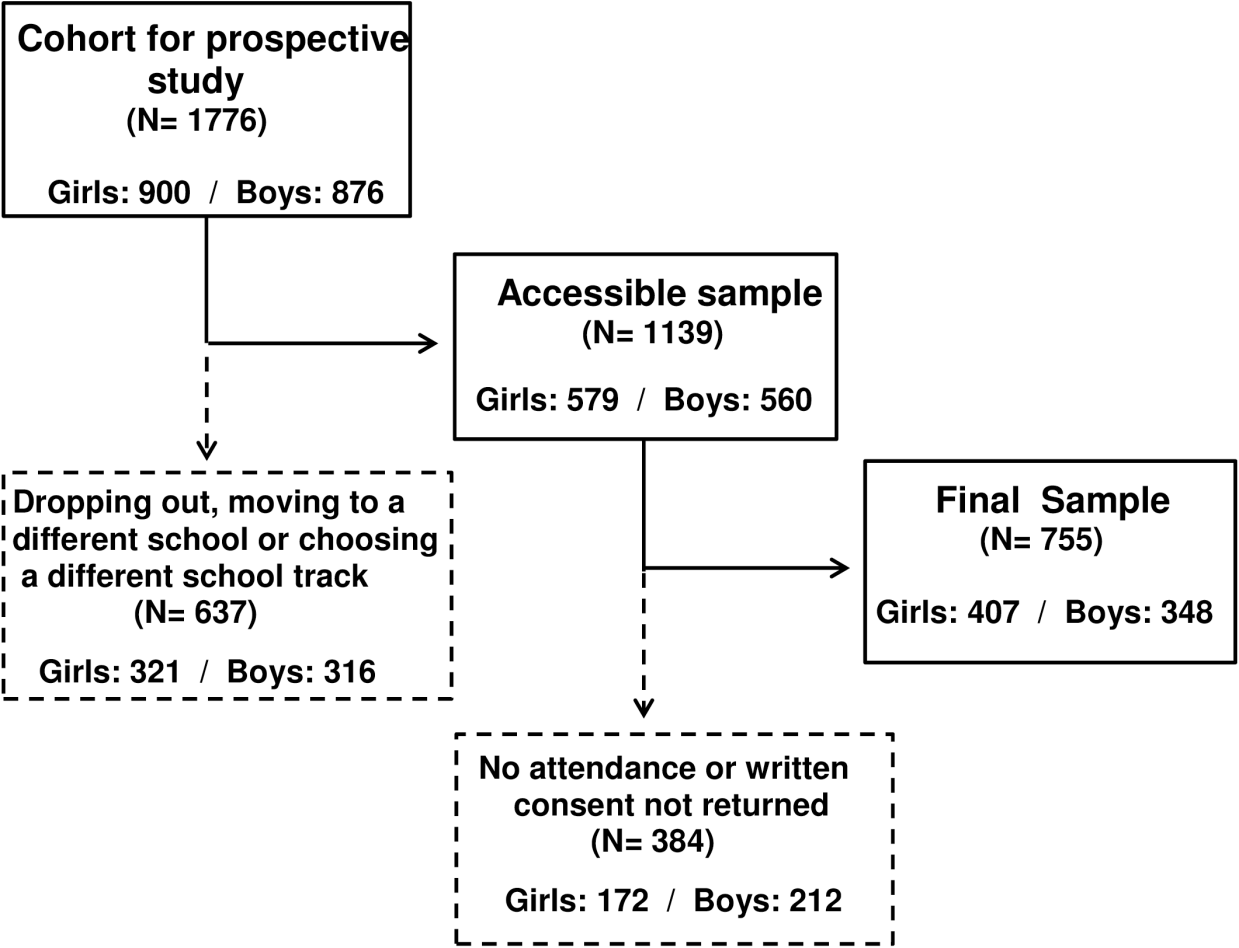
Flow diagram of the process for obtaining the final sample of the study.

Adolescents belonging to 13 secondary schools (7 state and 6 private) from six geographical areas of Spain participated in a prospective cohort study. The cohort was defined by those students aged 11–16 who participated in a previous cross-sectional representative study [30] in Fall 2010 (Wave I) and would still be enrolled in these schools three years later, when they reached the ages of 14–19 (Wave II) in Fall 2013. Resurveying the same variables to the same participants 3 years later allowed us to assess the potential changes in different adolescence stages. From the original cohort of 1776 students that participated in Wave I, 1139 remained as the potential or accessible cohort for Wave II since 637 (35.87%) failed to continue due to various reasons (dropping out from schooling, moving to a different school, or choosing a different school track). From the potential 1139 students, 345 did not attend to the data collection date for unknown reasons and 39 were not included in the final sample because never returned the written consent form. Therefore, 755 adolescents (348 boys and 407 girls) participated finally in Wave I and Wave II. It represents 66.29% of the accessible cohort which means 33.71% of attrition rate, similar to other longitudinal studies [31,32,33] and between a percentage range of 50.30% to 78.51% of three-year follow-up studies [34,35] (see Fig 1). Adolescents in this sample from Wave I showed a mean age of 12.92 (SD 0.89) ranging between 11 and 16 and from Wave II a mean age of 16.26 (SD 1.22) ranging between 14 and 19.

### Variables and instruments

The Seven-Day Physical Activity Recall (7D-PAR) was employed to assess the information related to different intensities of PA, low (LPA), moderate (MPA) and vigorous PA (VPA) [36]. For the present study, the average time of each PA intensity from the previous seven days was considered.

Adolescent Sedentary Activity Questionnaire (ASAQ) [37] was used to assess the time adolescents spent on daily sedentary behaviors. Variables regarding mobile phone usage were added in order to update the questionnaire. For the purposes of this paper SB was organized into three sedentary categories: academic activities (AA) (i.e. to study and do the homework with or without a computer); technological-based activities (TA) (e.g. watching TV/videos/DVDs and playing with computers, video consoles or mobile phones); and social-based activities (SA) (i.e. hanging out with friends and chatting via computers or cell phones).

ASAQ and 7DPAR have been proven reliable and valid [37,38] and used in previous longitudinal studies with heterogeneous samples in terms of weight status [39,40,41].

Finally, to establish weight status, Standardized BMI (sBMI) was calculated and recoded in three categorical variables, average weight, overweight and obese. To obtain these variables, participants’ height was measured to the nearest 0.1 cm using a stadiometer (MOD. 1-S-B, 6615) and weight was measured using a digital balance (Body Balance Comfort F5, Soehnle) to the nearest 0.1 kg, both in light weight clothing and with shoes removed. Standard procedures were used for all measurements [42]. BMI was recorded as weight (kg)/height^2^ (m^2^). sBMI was obtained from Z-scores for BMI for the study population using international age and sex-specific BMI cut-off points [43,44]. WHO AnthroPlus software was used to calculate scores (available on the WHO homepage: http://www.who.int/childgrowth/software/en/).

### Data analysis

Self-Organizing Map (SOM) analyses were used to detect and visualize changes of subjects’ characteristics between the first and second assessment. SOM was carried out with Matlab R2008a program (Mathworks Inc., Natick, USA) [45] and the SOM toolbox (version 2.0 beta) for Matlab. The process to obtain SOM can be explained in three steps.

The first step is the construction of a neuron network which size depends on the number of cases included in the analysis. The data matrix used as input has a length of 1510 cases (i.e. 755 subjects x 2 time moments). Using this data matrix, the size of the matrix was 20 x 10 neurons.

The second step was the initialization. During this process, a value or weight was assigned for each input variable to each of the neurons. SOM initialization was performed in two different ways: randomized and linear initialization.

The third step was the training of the SOM. The training process was used to modify the weights or values of the neurons assigned initially. Two different training algorithms were applied in this study (i.e. sequential and batch). Eq 1 was used to do the calculation during the neural network training:

$$w_{j}(n+1)=w_{j}(n)+\eta (n)\cdot h_{j,i(x)}(n)\cdot (x-w(n))$$ (1)

where *w*_*j*_ is the weight vector of the j^th^ neuron, η is the learning ratio, *h*_*j*,*i*_ is the neighborhood function and *x* is the input vector, *n* is the iteration and *i(x)* is the winning neuron of the x input vector. As can be seen, the changes of the neuronal weights depend on the difference between the starting weights and the input vector, the neighborhood function and the learning ratio. The neighborhood function allows the adaptation of the neural weights of the neurons that are far away from the winning neuron to decrease. For this study four different neighborhood functions were tested: i) gaussian, ii) cut gaussian, iii) Epanechicov and iv) Bubble.

The learning ratio had a high value during the first iterations and gradually decreased to very low values. At the beginning of the training, large changes in the neuron weight were produced, however their modification was more discreet as the process progressed.

The process described was repeated 100 times, because the result of the final analysis depended on some randomized processes (e.g. initialization and entry order of the input vector). By repeating the process 100 times the odds of finding the best solution to the problem were increased. Since we used two different training methods, four neighborhood functions and two initialization methods 1600 SOM were obtained (i.e. 100 x 2 x 4 x 2). The map with the least amount of errors when multiplying the quantization and topographical errors was selected.

**Fig 2:**
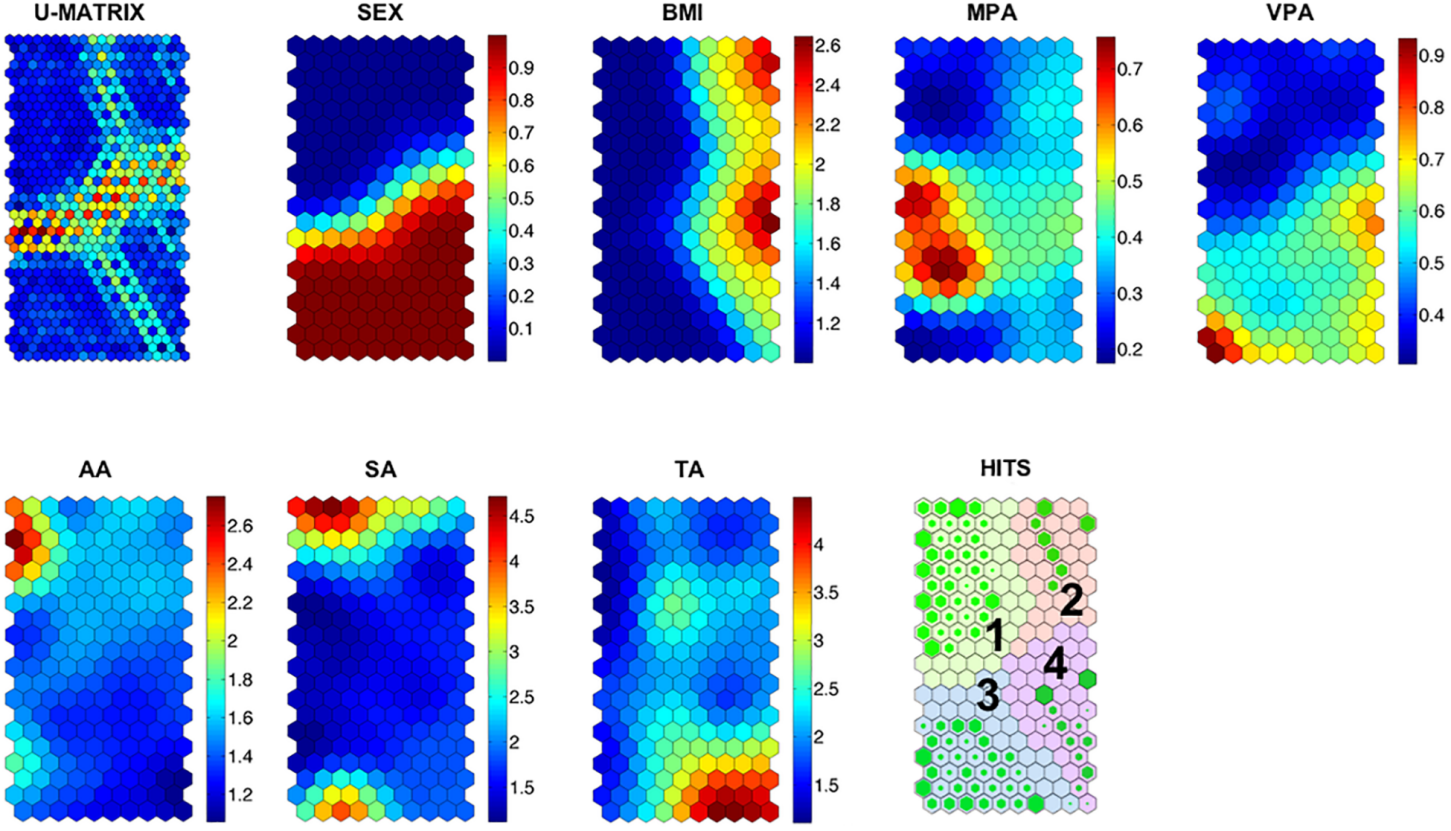
Component planes of the recorded variable. The Hits map can be seen in the right bottom corner. Empty cells show a lack of cases and the greener cells indicate a large accumulation of adolescents. Numbers in black (1–4) refer to quantitatively-analyzed clusters with results presented in Table 1. Rectangles on the right of each component map indicate the lower (bluish) and higher (reddish) values of each variable. Values for sex are 0 = boys and 1 = girls. Weight status is represented in categories (1 = normal weigh, 2 = overweight and 3 = obese). The rest of the variables are expressed in hours. In order to better understand the maps, it is important to note that participants included in every neuron (hexagon) are the same in all component planes. It should also be noted that each neuron represents the same subjects in all of the variable maps. Moreover, the neurons that are close to each other are more similar than those farther away. Therefore, x and y axis are related to the neuron placement in a multidimensional space. The x and y axes of each map do not represent any variable, but indicate the neuron position.

After SOM analysis, we used a k-mean method to classify the neurons in groups according to their characteristics, as reported by Davies and Bouldin [46]. The clusters found are shown in Fig 2 overlying the hit plane. Lastly, the Pearson correlation matrix between variables was calculated using the neuronal values. The type I error was established in less than 5% (α<0.05).

Moreover, the migrations of subjects from one cluster in Wave I to another in Wave II were quantified. The number of subjects per cluster that changed to different clusters three years after the first measurement was also calculated.

A classification tree was established using MatlabR2008a program (Mathworks Inc., Natick, USA) to discern the behavior modifications that facilitate the change of cluster. The difference between the post and pre-test for each of the PA and SB variables was included as input variables as well as the BMI at the beginning of the study. The objective of the classification tree was to distribute the subjects using the input variables in two different groups: those who changed clusters and those who did not. Thus, classification trees made it possible to determine which variables best discriminated between young people who changed from one cluster to another from those who remained in the same cluster in both assessments. Starting with the entire data pool, this method divides it into two groups using an independent explanatory variable. A cut-off point from the explanatory variable is determined (i.e., value of the explanatory variable used to split the node in two children nodes), meaning the cases that are below the threshold forms one group and the cases that are above the threshold form the other group. This process is repeated for each sub-group until all subjects are classified correctly. In the case of this study, CART algorithm with the Gini’s Diversity Index, as split criterion, is used to obtain the classification tree. Therefore, the classification tree divides youth according to key discriminating variables in order to classify all of the subjects according to their change of cluster (i.e., yes or no). Nevertheless, the tree was pruned to avoid having a ‘wide’ tree, with possibly a low external validity. The criteria used to choose the prone level was the highest that maintain the sensitivity and specificity above 80%.

## Results

Before describing the results, it is necessary to explain some aspects of the analysis that will help the reader to better interpret the SOM. At the time the analysis algorithm finished, all sample subjects (input) were placed in a given neuron (output). Thus, subjects were grouped in the same neuron with those colleagues with whom they shared more characteristics and they would always be placed in the same place (i.e. the same neuron). In this sense, each component plane (Fig 2) represents the value of one variable for the subjects allocated to each neuron. It should be noted that the subjects remain in the same neuron in all the component planes. For example, the upper left corner neuron contains subjects with high BMI, while they show medium to lower values in the rest of variables.

### SOM description and clusters

As a result of the SOM analysis, a final group of 7 maps corresponding to the 7 variables of our study emerged (see Fig 2). The gender map, for instance, showed that boys and girls were located on opposite sides (bluish top are girls and reddish bottom are boys). The visualization of the component plane, as a whole, permitted establishing reciprocal links or topological relationships among the maps. These relationships indicated that boys spent more time on MPA than girls, and this difference was sharper in VPA. Moreover, girls devoted more time than boys to academic activities (AA) and social-based activities (SA), especially a group of them. On the contrary, boys spent more time than girls on technological-based activities (TA), mainly a group of them. It is also worth noting that the overweight and obese boys and girls, located in the east zone of BMI map, devoted less time to AA, SA, TA, MPA and VPA than average-weight and underweight adolescents, although there was a small group of overweight and obese boys that spent a considerable time in VPA. Other topological relationships indicate that girls who spent more time on AA and SA devoted less time to MPA and VPA and boys who spent more time in TA devoted less time to MPA and moderate time in VPA.

These results were also supported by parametric analyses between the studied variables (see S1 Table). Particularly, significant negative associations emerged among academic activities with MPA and VPA (r = –0.38 and r = –0.44, respectively) and social-based activities with MPA (r = –0.60), while the association between technological-based activities and VPA turned out positive (r = 0.38). On the other hand, BMI was related positively with VPA (r = 0.18) but negatively with social-based activities (r = –0.15) and academic activities (r = –0.25).

Hits plane is placed at the bottom right corner of Fig 2. This map presented four identified clusters. Clusters 1 and 3 referred to overweight adolescents, and clusters 2 and 4 represented average-weight adolescents. Moreover, girls were grouped in clusters 3 and 4 and boys in clusters 1 and 2. Descriptive data and confidence intervals of each cluster are presented in Table 1.

**Table 1:** Descriptive statistics of each cluster (minutes/day).

	Boys overweight	Boys average-weight	Girls overweight	Girls average-weight
	Cluster 1 (n = 183)	Cluster 2 (n = 183)	Cluster 3 (n = 495)	Cluster 4 (n = 201)
	*M*(SD)	*M*(SD)	*M*(SD)	*M*(SD)
	[95% CI]	[95% CI]	[95% CI]	[95% CI]
MPA	0.35(0.16)	0.36(0.02)	0.42(0.17)	0.42(0.04)
	[0.31–0.38]	[0.35–0.37]	[0.38–0.46]	[0.40–0.43]
VPA	0.37(0.04)	0.40(0.06)	0.60(0.09)	0.64(0.06)
	[0.37–0.39]	[0.38–0.42]	[0.58–0.63]	[0.61–0.65]
AA	1.66(0.32)	1.54(0.05)	1.38(0.15)	1.31(0.10)
	[1.58–1.74]	[1.53–1.56]	[1.34–1.42]	[1.28–1.34]
SA	2.10(1.04)	1.90(0.51)	1.90(0.68)	1.66(0.14)
	[1.86–2.36]	[1.73–2.06]	[1.72–2.07]	[1.61–1.70]
TA	1.87(0.42)	1.93(0.20)	2.60(0.88)	2.36(0.70)
	[1.77–1.97]	[1.86–2.00]	[2.37–2.82]	[2.14–2.57]

Abbreviations: M(SD), Mean (Standard deviation); CI (Confidence Interval); MPA, moderate physical activity; VPA, vigorous physical activity; AA, academic activities; SA, social-based activities; TA, technological-based activities

### Cluster migrations

**Fig 3:**
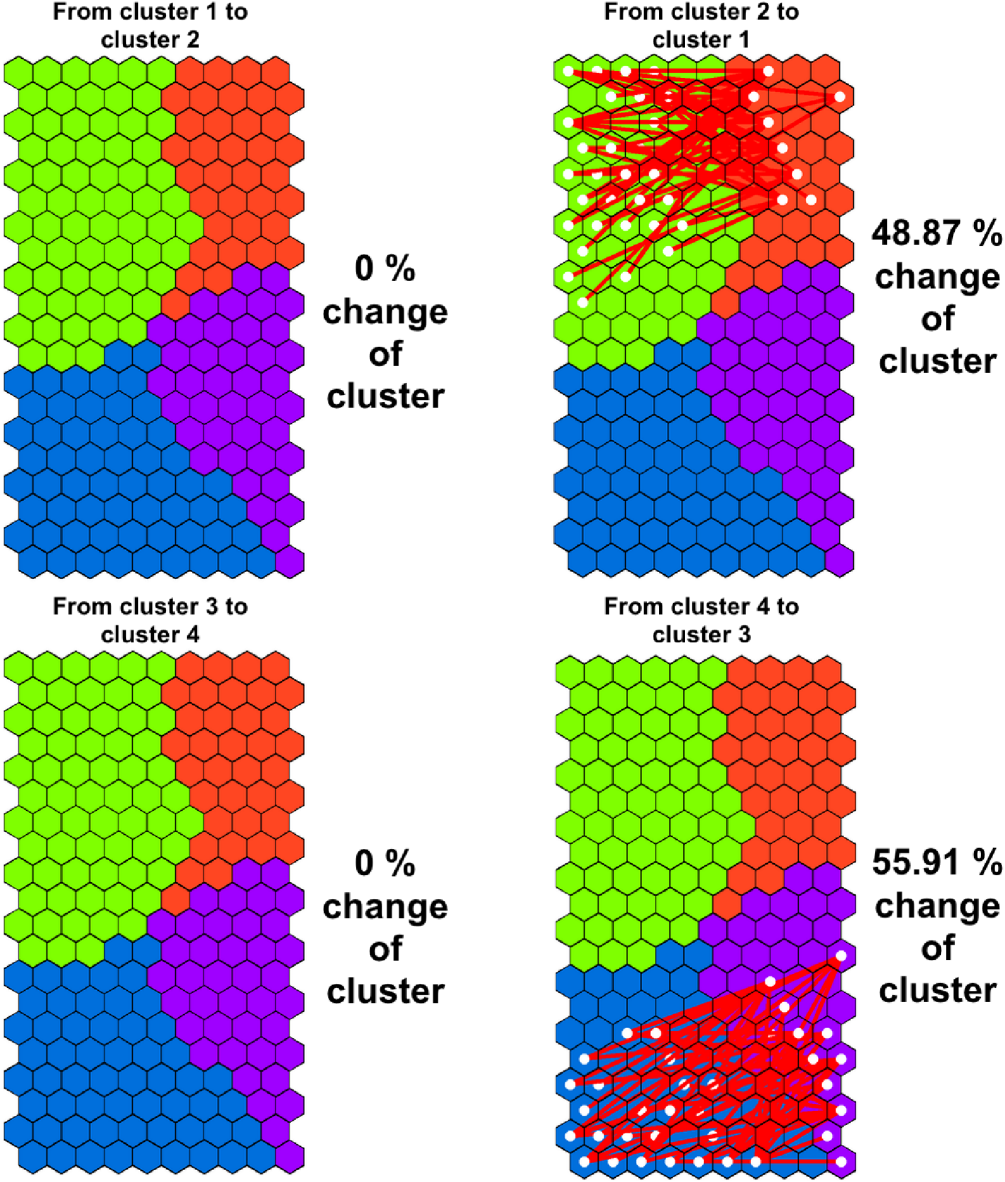
Participants’ migrations between clusters of different weight status.

The trajectories followed by the participants during the period of time between the two measures are showed in Fig 3. It is remarkable that some subjects placed in clusters 4 and 2 (overweight and obese zones) migrated to the opposite side (average-weight zones) 3 years later.

Specifically, the changes in adolescents’ BMI indicate that 103 girls from cluster 2 (48.9% of the cluster subjects) reached cluster 1, and 98 boys from cluster 4 (55.9% of the cluster subjects) migrated to cluster 3 after this time period. In other words, around 22.66% of the adolescents’ sampled had changed from the overweight/obese zone to the average-weight status zone after three years. No changes were observed from cluster 3 to cluster 4 nor from cluster 1 to cluster 2.

### Changes towards a different weight cluster

**Fig 4:**
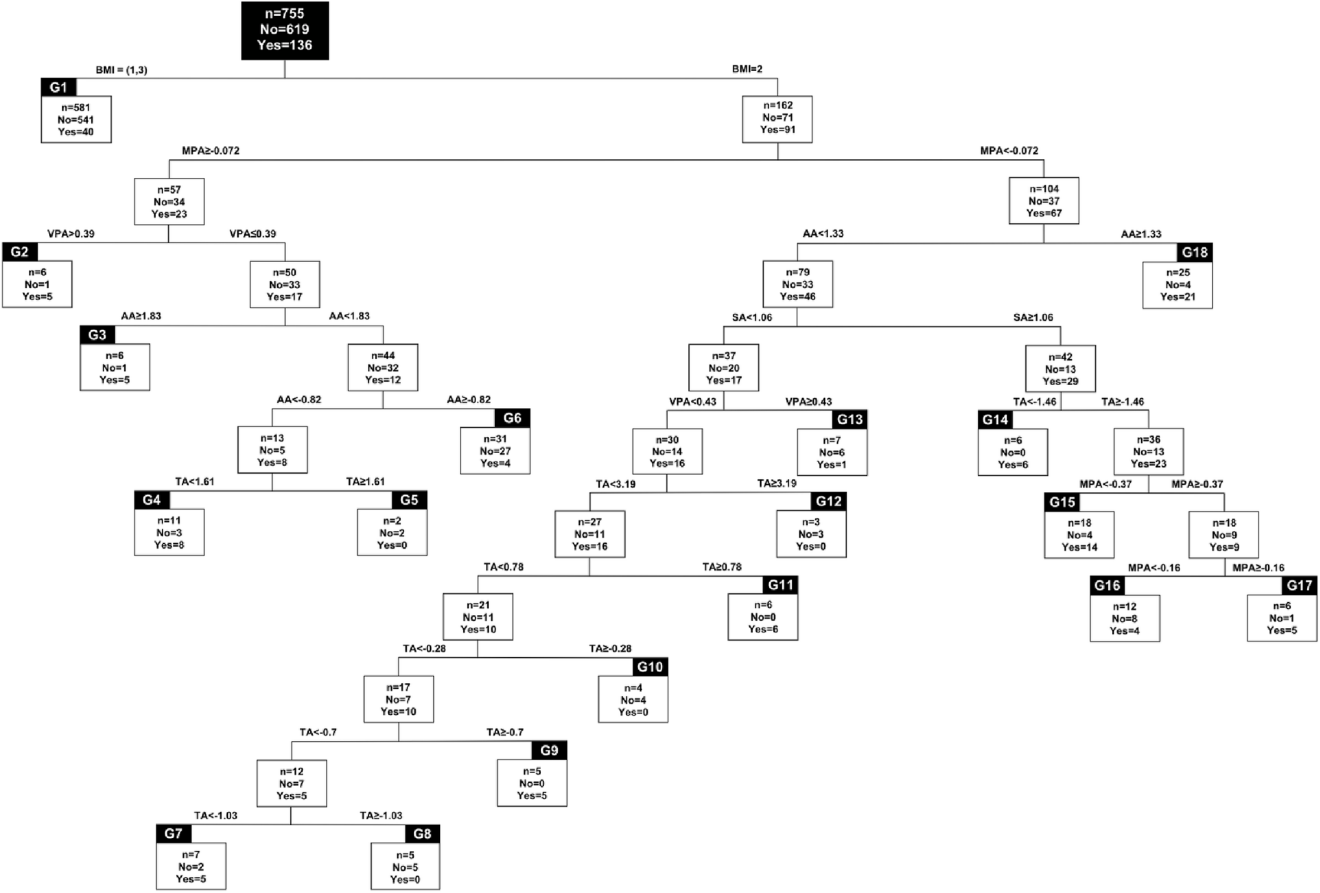
Classification tree.

A classification tree was carried out to exhibit adolescents’ groups of change in their weight status towards a different weight cluster (Fig 4). All changes corresponded to migrations towards a lower weight cluster. In particular, ten of the eighteen groups identified comprised the majority of participants who had changed. Although the behavioral patterns of these groups are presented in Table 2, in particular, three were highlighted for diverse reasons. The accuracy of the classification using the decision tree was of 91%.

**Table 2:** Behavioral characteristics and percentages of change in overweight subjects who switched to a normal weight cluster.

	Changes in PA (hours/day)	Changes in SB (hours/day)	% of cluster change
**Group 2**	MPA: Increased or decreased a maximum of 0.72		83.3
	VPA: Increased more than 0.39		
**Group 3**	MPA: Increased or decreased a maximum of 0.72	AA: Increased by at least 1.83	83.3
	VPA: Decreased or increased a maximum of 0.39		
**Group 4**	MPA: Increased or decreased a maximum of 0.72	AA: Decreased by at least 0.82	72.7
	VPA: Decreased or increased a maximum of 0.39	TA: Decreased or increased a maximum of 1.61	
**Group 7**	MPA: Decreased by at least 0.72	AA: Decreased or increased a maximum of 1.33	71.4
	VPA: Decreased or increased a maximum of 0.43	SA: Decreased or increased a maximum of 1.06	
		TA: Decreased at least 1.03	
**Group 9**	MPA: Decreased by at least 0.72	AA: Decreased or increased a maximum of 1.33	100
	VPA: Decreased or increased a maximum of 0.43	SA: Decreased or increased a maximum of 1.06	
		TA: Decreased between 0.28 and 0.7	
**Group 11**	MPA: Decreased by at least 0.72	AA: Decreased or increased a maximum of 1.33	100
	VPA: Decreased or increased a maximum of 0.43	SA: Decreased or increased a maximum of 1.06	
		TA: Increased between 0.78 and 3.19	
**Group 14**	MPA: Decreased by at least 0.72	AA: Decreased or increased a maximum of 1.33	100
		SA: Increased by at least 1.06	
		TA: Decreased by at least 1.46	
**Group 15**	MPA: Decreased by at least 0.37	AA: Decreased or increased a maximum of 1.33	77.7
		SA: Increased by at least 1.06	
		TA: Increased or decreased a maximum of 1.46	
**Group 17**	MPA: Decreased between 0.16 and 0.72	AA: Decreased or increased a maximum of 1.33	83.3
		SA: Increased by at least 1.06	
		TA: Increased or decreased a maximum of 1.46	
**Group 18**	MPA: Decreased by at least 0.72	AA: Increased by at least 1.33	18.84

Note: PA (Physical activity); MPA (Moderate physical activity); VPA (Vigorous physical activity); SB (Sedentary behaviors); AA (Academic activities); SA (Social-based activities); TA (Technological-based activities)

Group number 18 represents the highest percentage of the adolescents’ entire sample (i.e., 15.4%) who changed from an overweight/obese cluster to an average-weight cluster out of the total adolescents who changed clusters. These participants decreased their MPA and their AA in at least 0.72 and 1.33 hours/day respectively.

Group number 11 exemplifies the group in which all of the adolescents (n = 6) changed from an overweight/obese cluster to an average-weight cluster. These participants decreased their MPA in at least 0.37 hours/day, either decreased their AA or increased it by a maximum of 1.33 hours/day, increased their SA by at least 1.06 hours/day and increased their TA or decreased it by a maximum of 1.46 hours.

Finally, group 4 represents the group which showed the lowest percentage of change (72.7%, i.e., 8 subjects from 11). These participants either increased or decreased their MVP, but by a maximum of 0.72 hours. They also increased or decreased their VPA by a maximum of 0.39 hours, decreased their AA by at least 0.82 hours and increased or decreased their TA by a maximum of 1.61 hours.

## Discussion

This study examines longitudinal changes in PA, SB and BMI in Spanish adolescents, specifically focused on those who migrate towards a different weight status or category. Previous longitudinal studies have studied these changes by using a linear statistical method, but this is the first time that a clustering SOM analysis is performed in a longitudinal study to examine this triumvirate of variables and the migrations between clusters. This paper is also innovative in using a classification tree to focus on the behavioral characteristics of the groups of adolescents who migrated towards a different weight status.

The main longitudinal results from cluster migrations indicate that an important group of adolescents (48.9% of girls and 55.9% of boys) that were classified as overweight or obese in Wave I changed to average-weight in Wave II. Therefore, 22.66% of the adolescents from the total sample changed their weight cluster to a lower category and none of them changed in the opposite direction. Although this is a surprising result, it is necessary to pay attention that some participants may increase or decrease their weight three years later but their original behaviors have not changed enough to move towards another cluster. Even so, these changes of cluster move towards a reduction of weight while many studies refer to the increase in the prevalence of overweight and obesity in adolescents [2,47]. A possible explanation of this counterintuitive result refers to a biological or developmental reason due to the growing ages of the sample [48]. The behavioral characteristics of the groups of adolescents that move into a lower weight cluster (see Table 2) do not enlighten us about the longitudinal results. These characteristics do not support the hypothesis that the change to a lower weight cluster is a result of an increase in time devoted to PA or a decrease in time spent on SB [14,15,29]. The longitudinal results of this paper are closer to the studies that report PA exerts a weak influence on overweight and obesity [23], as well as other studies that found no clear effect of academic activities, social-based activities and technological-based activities on weight [25]. Therefore, the results suggest that there may be other ways of changing to a lower-weight status in adolescents apart from those in which PA and SB are involved.

The results about interrelationships among PA, SB and BMI, obtained from the visualization of SOM, are consistent with those studies that indicate boys are more active and tend to be less sedentary than girls [26]. Nevertheless, in terms of SB, this paper is in line with previous studies which show that boys devote more time to TA, such as computer and videogames, than girls [28], and that girls spend more time on SA, such as meeting with friends or using mobile phones for social interaction than boys [8]. The results also show a moderate competing effect between SB and PA, especially in girls, since the groups of girls who spend the highest amount of time on AA and SA are the same as those who devote less time to MPA and VPA, in a similar vein as a recent SOM analysis that focused on girls [49]. Finally, overweight and obese adolescents devoted less time to AA, SA, TA, MPA and VPA than average-weight and underweight adolescents, although there is a small group of overweight and obese boys that spent a considerable amount of time in VPA. These topological links suggest, as do many other studies [3,6], that overweight and obesity are negatively associated with PA, although a small group of overweight/obese adolescents shows a positive relationship with VPA, probably due to an assumed necessity to increase time spent in VPA to lose weight [14].

There are some limitations that should be considered when interpreting the results from this study. The first limitation emerges from the self-reported measures of behavioral variables. Due to previous evidence concluding that subjective methods of data collection, such as questionnaires, may overestimate or underestimate specific information reported by teenagers [50], some concern about the reliability and accuracy of the data arises. However, these instruments were previously proven reliable and valid [37,38] and have been used in several international studies [30,49,51]. Furthermore, measurement errors were minimized by employing standardized protocols and guidelines.

A second limitation of this research study is its modest capability to explain some of the studied phenomena. Adolescents’ changes towards a lower weight status are not explained by longitudinal changes in PA and SB. A plausible explanation for these limitations might be the complexity of the triumvirate relationships among PA, SB and weight status. Furthermore, the potential effects of several variables that were not regarded in this research such as sleep time, school ethos, peer acceptance or rejection, motivation toward physical activity or caloric intake should be considered.

Despite the aforementioned limitations, this study exhibits several strengths to examining longitudinal changes in PA, SB and BMI in Spanish adolescents, focusing especially on those adolescents who migrate to a different weight status. Results from this study highlights that almost a quarter of the adolescents from the total sample changed their weight status to a lower category and none of them changed in the opposite direction. Moreover, characteristics that stem from the innovative classification tree do not support the idea that PA and SB exert a substantial effect on overweight and obesity. Therefore, these results are useful for exploring other ways to change to a lower-weight status in adolescents apart from those in which PA and SB are involved. The biological and developmental factors associated to the growing ages of adolescents become particularly important.

## Supporting information

S1 Table Pearson correlation coefficients among variables. (DOCX)
