# Supplementary material for: Longitudinal changes in physical activity, sedentary behavior and body mass index in adolescence: Migrations towards different weight cluster
Source: PLoS One. 2017 Jun 21;12(6):e0179502. doi: 10.1371/journal.pone.0179502 (PMC5479538; doi:10.1371/journal.pone.0179502)
Supplement: S1 Table — (DOCX) [file pone.0179502.s001.docx]

|  |  | Sex | sBMI | MPA | VPA | AA | SA | TA |
| --- | --- | --- | --- | --- | --- | --- | --- | --- |
| Sex |  | 1 |  |  |  |  |  |  |
|  |  | [1.00–1.00] |  |  |  |  |  |  |
| sBMI |  | 0.06 | 1 |  |  |  |  |  |
|  |  | [-0.07–0.20] | [1.00–1.00] |  |  |  |  |  |
| MPA |  | 0.25* | 0.05 | 1 |  |  |  |  |
|  |  | [0.11–0.37] | [-0.09–0.18] | [1.00–1.00] |  |  |  |  |
| VPA |  | 0.90* | 0.18* | 0.05 | 1 |  |  |  |
|  |  | [0.88–0.93] | [0.04–0.31] | [-0.09–0.19] | [1.00–1.00] |  |  |  |
| AA |  | -0.56* | -0.25* | -0.38* | -0.44* | 1 |  |  |
|  |  | [-0.65– -0.47] | [-0.37– -0.11] | [-0.49– -0.26] | [-0.55– -0.32] | [1.00–1.00] |  |  |
| SA |  | -0.17* | -0.15* | -0.60* | -0.03 | 0.34* | 1 |  |
|  |  | [-0.30– -0.03] | [-0.28– -0.01] | [-0.68– -0.51] | [-0.17– 0.11] | [0.22–0.46] | [1.00–1.00] |  |
| TA |  | 0.50* | 0.01 | -0.08 | 0.38* | -0.60* | -0.03 | 1 |
|  |  | [0.38–0.59] | [-0.13–0.14] | [-0.21–0.06] | [0.26–0.49] | [-0.68– -0.50] | [-0.17–0.10] | [1.00–1.00] |

**S1 Table. Pearson correlation coefficients among variables.**

Data are expressed as correlation coefficients [95% confidence intervals]. *Significant correlation to 0.01 level (bilateral). Abbreviations: sBMI, standardized body mass index; MPA, moderate physical activity; VPA, vigorous physical activity; AA, academic activities; SA, social-based activities; TA, technological-based activities.
